# Whitman’s Orthopedic Surgery

**Published:** 1901-10

**Authors:** 


WHITMAN'S ORTHOPEDIC SURGERY. For Students, Practitioners and Specialists. By ROYAL WHITMAN, M. D, Adjunct Professor of Orthopedic Surgery, New York Polyclinic; Instructor in Orthopedic Surgery in the College of Physicians and Surgeons, and Chicf of Orthopedic Department in the Vanderbilt Clinic, New York. In one 8vo volume of 642 pages, with 447 illustrations. Philadelphia and New York: Lea Brothers & Co. 1901. [Price, cloth, $5.50 net.]

It is with unusual interest and expectation that we have examined this surgery of Royal Whitman, whose past experimental work and original research in his specialty are known to everyone. It must be confessed that our expectations are gratified. The numerous original illustrations alone afford evidence of exhaustive and patient endeavor.

In its general arrangement and order the book resembles Bradford and Lovett's orthopedic surgery. The opening chapter on Pott's disease is exhaustive, and as elsewhere the methods of examination and treatment are fully dealt with while pathological details receive less attention. In the treatment stress is laid on anterior support for the shoulders, to prevent forward bending of the spine when the disease lies in the upper and middle dorsal region. Cabot's operation is given in full, but a decided opinion for or against this procedure is withheld. In discussing lateral curvature, exercises and forcible correction without the use of jackets or supports are advocated. Teschner's exercises and the series in use at the hospital for ruptured and crippled, are outlined with the aid of illustrations.

The American treatment of hip-disease by traction and the English treatment by fixation are distinguished and their relative efficiency discussed. The author has attempted to combine the two methods, and there is now in use at the hospital a traction brace, which has given satisfaction, with lateral thoracic bar and metal band encircling the chest. The articles on congenital dislocation of the hip and coxa vara are especially good. A general description of the foot, its anatomy, arches and functions is given in which the foot is regarded as a machine,—a valuable introduction to the study of the weak foot and the club foot. Altogether, we can heartily recommend this surgery as a standard text-book and reference work. The binding, illustrations and printing are most satisfactory. P. LeB.

